# Supplementary figures and images for: Alu elements in primates are preferentially lost from areas of high GC content
Source: PeerJ. 2013 May 21;1:e78. doi: 10.7717/peerj.78 (PMC3661076; doi:10.7717/peerj.78)

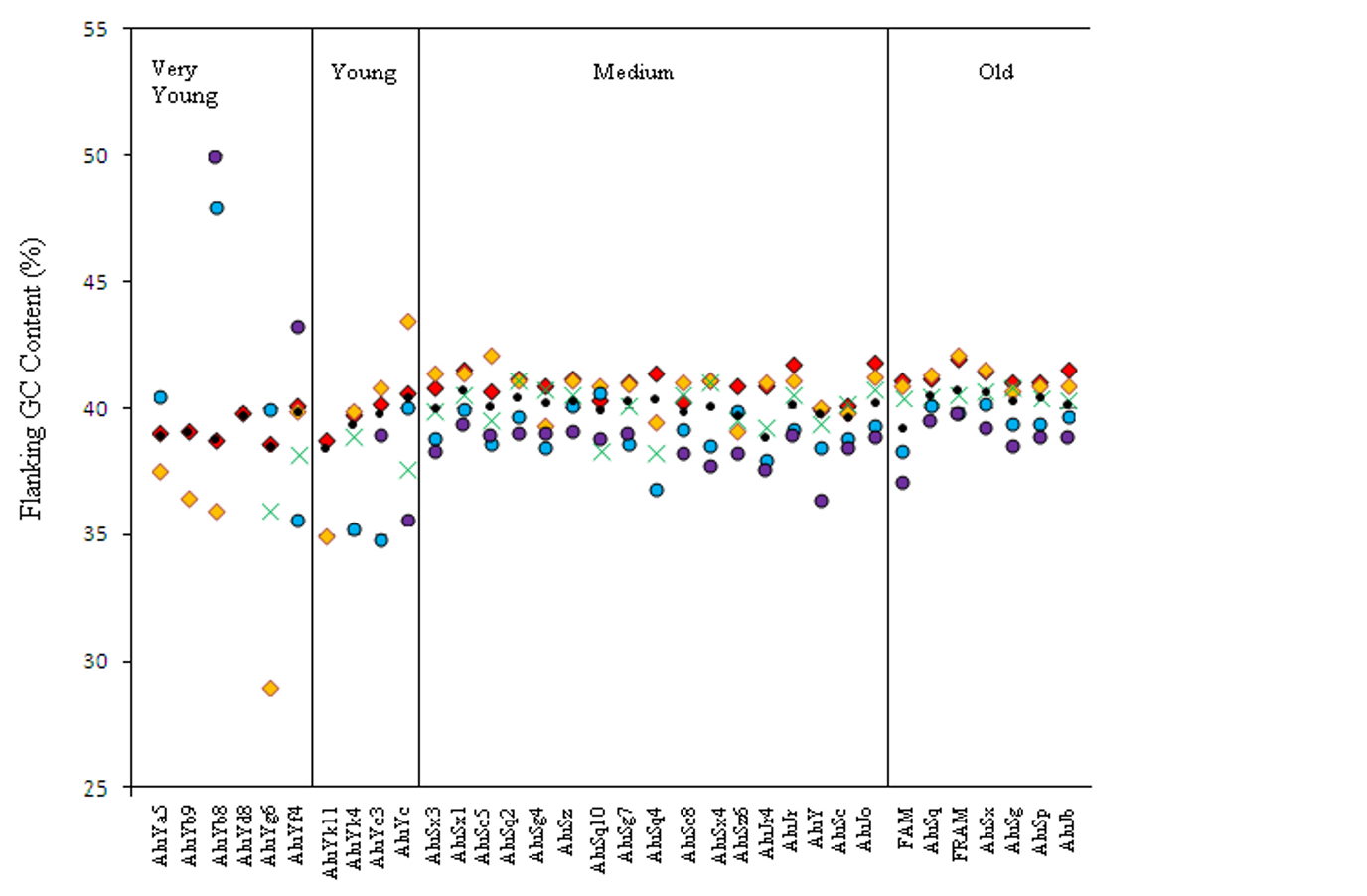

Supplement: Figure S1 — Mean GC content of Alu flanking regions in each subfamily, classified by presence in the primate species which is most distant from humans. Elements with an unclear evolutionary history, for example ones found in humans and gorillas, but not chimpanzees, are included in this analysis, but excluded from the analysis shown in Fig. 4. [file peerj-01-78-s001.png]
