# Supplementary material for: Alu elements in primates are preferentially lost from areas of high GC content
Source: PeerJ. 2013 May 21;1:e78. doi: 10.7717/peerj.78 (PMC3661076; doi:10.7717/peerj.78)
Supplement: Table S1 — The number of elements found in each species grouping (human, human and chimp, etc.) for each subfamily. Elements with unclear evolutionary lineages (e.g. found in gorillas and humans, but not in chimpanzees) have been removed, as in the analysis shown in Fig. 4. [file peerj-01-78-s002.docx]

|  | Human | Chimp | Gorilla | Orangutan | Macaque | Total |
| --- | --- | --- | --- | --- | --- | --- |
| AluYa5 | 133 | 4 | 0 | 4 | 0 | 141 |
| AluYb9 | 31 | 1 | 0 | 0 | 0 | 32 |
| AluYb8 | 217 | 3 | 0 | 0 | 0 | 220 |
| AluYd8 | 17 | 0 | 0 | 0 | 0 | 17 |
| AluYg6 | 42 | 1 | 1 | 0 | 0 | 44 |
| AluYf4 | 70 | 13 | 6 | 2 | 0 | 91 |
| AluYk11 | 4 | 1 | 0 | 0 | 0 | 5 |
| AluYk4 | 62 | 10 | 18 | 23 | 0 | 113 |
| AluYc3 | 27 | 4 | 2 | 1 | 0 | 34 |
| AluYc | 131 | 20 | 16 | 23 | 1 | 191 |
| AluSx3 | 1604 | 169 | 135 | 267 | 630 | 2805 |
| AluSx1 | 5846 | 666 | 475 | 1053 | 2942 | 10982 |
| AluSc5 | 345 | 33 | 28 | 78 | 125 | 609 |
| AluSq2 | 3024 | 332 | 294 | 545 | 1395 | 5590 |
| AluSg4 | 379 | 42 | 29 | 55 | 134 | 639 |
| AluSz | 5028 | 561 | 460 | 883 | 3057 | 9989 |
| AluSq10 | 90 | 14 | 6 | 12 | 19 | 141 |
| AluSg7 | 466 | 47 | 23 | 67 | 118 | 721 |
| AluSq4 | 74 | 10 | 4 | 12 | 26 | 126 |
| AluSc8 | 1189 | 139 | 85 | 175 | 287 | 1875 |
| AluSx4 | 307 | 35 | 25 | 65 | 119 | 551 |
| AluSz6 | 2032 | 212 | 208 | 406 | 1478 | 4336 |
| AluJr4 | 699 | 75 | 71 | 198 | 773 | 1816 |
| AluJr | 2844 | 305 | 318 | 687 | 2683 | 6837 |
| AluY | 6748 | 968 | 586 | 1129 | 48 | 9479 |
| AluSc | 1896 | 208 | 154 | 369 | 658 | 3285 |
| AluJo | 2568 | 227 | 302 | 611 | 2233 | 5941 |
| FAM | 278 | 30 | 32 | 69 | 276 | 685 |
| AluSq | 1156 | 142 | 110 | 245 | 529 | 2182 |
| FRAM | 357 | 45 | 41 | 79 | 348 | 870 |
| AluSx | 6206 | 680 | 577 | 1111 | 3078 | 11652 |
| AluSg | 2412 | 266 | 201 | 415 | 902 | 4196 |
| AluSp | 3061 | 295 | 269 | 545 | 710 | 4880 |
| AluJb | 5762 | 587 | 659 | 1212 | 4611 | 12831 |
