# Supplementary material for: Alu elements in primates are preferentially lost from areas of high GC content
Source: PeerJ. 2013 May 21;1:e78. doi: 10.7717/peerj.78 (PMC3661076; doi:10.7717/peerj.78)
Supplement: Table S2 — The table contains ‘Very young’ human elements for which orthologs can be found in other primate species. The Alu subfamily annotation for each ortholog is shown and annotations which are different from that found in humans are shown in bold. In the “Species” column, “X” indicates the absence, and “1” the presence of the element in the species macaque, orang utan, gorilla, chimpanzee and human. [file peerj-01-78-s003.docx]

| **Element** | **Species** | **Macaque** | **Orang-utan** | **Gorilla** | **Chimpanzee** |
| --- | --- | --- | --- | --- | --- |
| AluYa5_chr2_106875414-106876686 | XXX11 | N/A | N/A | N/A | AluYa5 |
| **AluYa5_chr2_17421036-17422340** | X1111 | N/A | **AluY** | AluYa5 | **AluY** |
| **AluYa5_chr3_130083613-130084919** | X1111 | N/A | **AluY** | **AluY** | **AluY** |
| **AluYa5_chr3_982099-983408** | XXX11 | N/A | N/A | N/A | **AluSp** |
| **AluYa5_chr3_44747765-44748988** | XXX11 | N/A | N/A | N/A | **AluSq2** |
| **AluYa5_chr3_1778263-1779559** | XXX11 | N/A | N/A | N/A | **AluSx4** |
| **AluYa5_chr4_103372213-103373520** | X1111 | N/A | **AluY** | **AluY** | **AluY** |
| **AluYa5_chr4_115134269-115135467** | X1111 | N/A | **AluY** | AluYa5 | AluYa5 |
| **AluYb8_chr3_21643506-21644824** | XXX11 | N/A | N/A | N/A | **FRAM** |
| **AluYb8_chr3_25030760-25032089** | XXX11 | N/A | N/A | N/A | **AluSc** |
| **AluYb8_chr4_65597012-65598331** | XXX11 | N/A | N/A | N/A | **AluY** |
| **AluYb9_chr3_2311142-2312432** | XXX11 | N/A | N/A | N/A | **AluSc** |
| AluYf4_chr1_108364495-108365830 | XX111 | N/A | N/A | AluYf4 | AluYf4 |
| AluYf4_chr1_207000254-207001577 | XXX11 | N/A | N/A | N/A | AluYf4 |
| AluYf4_chr1_1215063-1216362 | XXX11 | N/A | N/A | N/A | AluYf4 |
| **AluYf4_chr2_143640253-143641566** | X1111 | N/A | **AluY** | AluYf4 | AluYf4 |
| AluYf4_chr2_102024166-102025465 | XXX11 | N/A | N/A | N/A | AluYf4 |
| AluYf4_chr2_123313894-123315196 | XXX11 | N/A | N/A | N/A | AluYf4 |
| AluYf4_chr2_34917353-34918543 | XXX11 | N/A | N/A | N/A | **AluYc** |
| AluYf4_chr2_70692867-70694176 | XX111 | N/A | N/A | AluYf4 | AluYf4 |
| AluYf4_chr2_45713085-45714401 | XX111 | N/A | N/A | **AluY** | **AluY** |
| **AluYf4_chr3_154622828-154624127** | X1111 | N/A | **AluY** | AluYf4 | AluYf4 |
| AluYf4_chr3_61068482-61069781 | XXX11 | N/A | N/A | N/A | AluYf4 |
| AluYf4_chr3_162623712-162625002 | XXX11 | N/A | N/A | N/A | AluYf4 |
| AluYf4_chr3_196579423-196580641 | XXX11 | N/A | N/A | N/A | AluYf4 |
| AluYf4_chr3_156115600-156116897 | XX111 | N/A | N/A | AluYf4 | AluYf4 |
| AluYf4_chr4_114097777-114099035 | XX111 | N/A | N/A | AluYf4 | AluYf4 |
| AluYf4_chr4_189686328-189687628 | XX111 | N/A | N/A | AluYf4 | AluYf4 |
| AluYf4_chr4_112097376-112098690 | XXX11 | N/A | N/A | N/A | AluYf4 |
| AluYf4_chr22_21878918-21880213 | XXX11 | N/A | N/A | N/A | AluYf4 |
| AluYf4_chr22_20434950-20436245 | XXX11 | N/A | N/A | N/A | AluYf4 |
| AluYf4_chr22_21763252-21764547 | XXX11 | N/A | N/A | N/A | AluYf4 |
| AluYf4_chr22_18502073-18503378 | XXX11 | N/A | N/A | N/A | AluYf4 |
| **AluYg6_chr3_19689910-19691212** | XXX11 | N/A | N/A | N/A | **AluSx3** |
| AluYg6_chr3_113982570-113983879 | XX111 | N/A | N/A | AluYg6 | AluYg6 |
